# Graphene Enhances the Loading Capacity and Lubrication Performance of Ionic Liquids: A Molecular Dynamics Study

**DOI:** 10.3390/ma16144942

**Published:** 2023-07-11

**Authors:** Haodong Jiang, Yaoze Wang, Zhipeng Xiong, Runhua Zhou, Linyan Yang, Lichun Bai

**Affiliations:** 1 Key Laboratory of Traffic Safety on Track (Central South University), Ministry of Education, School of Traffic & Transportation Engineering, Central South University, Changsha 410075, China; 2 Energy Research Institute @ NTU (ERI@N), Nanyang Technological University, 50 Nanyang Avenue, Singapore 639798, Singapore; 3 School of Resources and Environmental Engineering, East China University of Science and Technology, Shanghai 200237, China

**Keywords:** graphene, ionic liquid lubricants, molecular dynamics simulation, diamond-like carbon films, lubrication

## Abstract

Ionic liquid (IL) combined with graphene additives have garnered extensive attention in the field of high-performance lubricating materials. However, the ambiguous mechanism of graphene influencing the load-carrying and anti-wear capacity of ILs needs further study. In this work, friction simulation shows that adding graphene causes friction coefficient to reduce by up to 88% compared with pure ILs, but lubrication performance is lost due to the destruction of graphene under high stress. Meanwhile, multilayer graphene has better friction-reducing performance and friction durability as compared to the monolayer structure, which is attributed to the easy-shear property and the reduction in the percentage of high tensile stress sites in multilayer graphene structure. In addition, it was found that excessively thick ILs film would form a three-body abrasive wear structure with graphene, which accelerated the structural destruction of graphene and caused a decline in its tribological properties. It is believed these findings can be valuable for designing of high-performance lubricating oil for practical engineering.

## 1. Introduction

Since the initial report published in 2001 [1], ionic liquid (IL) lubricants have become an important research object in the field of tribology and high-performance lubricating material due to their extremely negligible volatility, thermal stability and chemical stability [2,3,4,5,6,7,8]. According to previous research, it has been supposed that ILs can protect the substrate surface through the formation of boundary lubrication film [9,10,11,12]. However, under high loads, the relatively poor anti-wear performance and load-carrying capacity of pure ionic liquid limit its wide application in the field of tribology [13,14]. In recent years, researchers found that the drawbacks of ILs lubricants can be improved by adding specific additives and load-carrying phase to ILs [15,16,17,18,19]. One such additive is the emerging two-dimensional materials, such as graphene, which exhibits exceptional mechanical and ultra-low friction coefficients. In addition, multilayer graphene even shows low average friction with no oscillation due to the thermal escape motion [20,21,22]. Consequently, it has become ideal addition for ILs and can significantly improve the lubrication performance [23,24,25].

Recently, extensive effort has been devoted to investigating the tribological properties of composite lubricants based on ILs and graphene. Functionalized multilayer graphene as additives can prominently enhance the anti-wear and friction-reducing ability of IL film under high vacuum, due to the lubrication synergy of ILs and multilayer graphene [26]. Qi et al. found that the friction coefficient and anti-wear life were greatly improved with graphene as additives, ascribing to the improved load-carrying capacity and second lubricating phase [27]. Saurín et al. found that the addition of monolayer graphene increases friction and wear rates due to the agglomerates of graphene in the epoxy resin–stainless steel interface, but they decrease with the addition of the same proportion of dispersed multilayer graphene [28]. Moreover, the introduction of ILs solves the problem of poor dispersibility of graphene and the formation of lubricating film has a positive effect of friction reduction [29].

The synergistic lubrication mechanism of graphene as additives for ILs has been studied from various aspects of tribochemical reactions and enhancing load-carrying capacity. However, since the scale of graphene as a low-dimensional material has entered the nanoscale, it is difficult to elucidate the microstructure and frictional properties of nano-additives only through experimental studies. Meanwhile, macroscopic experiments cannot observe the synergistic lubrication process as well as the structural changes of additives, and cannot obtain accurate lubrication mechanisms to efficiently guide the optimization of experimental processes. Therefore, the mechanism of graphene molecules as additives affecting tribology behavior of ILs is still unclear. The computer simulation method helps us to gain insight into the friction mechanism from the atomic scale. Hence, it has been widely used to observe the mechanism in tribology field.

In this work, a molecular dynamics simulation (MD) approach is used to investigate the synergistic lubrication mechanism based on ILs and graphene. Diamond-like carbon (DLC) is employed as the friction substrate in this research due to its antifriction performance. It was found that their excellent lubrication performance, which has a significant dependence on the application environment, are dementedly influenced by the sp3-to-sp2 ratio [30]. And the effect of adhesion between DLC substrates can make the friction interfacial evolution easy to analyze [31]. Therefore, it has been widely used in the research of ILs lubrication field. This research focuses on the effects of factors, such as normal stress, amounts of graphene, amounts of ionic liquids, surface roughness, and sliding velocity. Most importantly, the evolution of the interfacial structure as well as the structural evolution of the additives was systematically investigated. The results show that the frictional performance of the composite lubricant is highly dependent on the laminar structure of graphene, normal stress, and the thickness of the lubricating film. This study helps to guide the experimental procedure of efficient lubrication and accelerate the development of high-performance fluid lubricants.

## 2. Modeling

**Figure 1:**
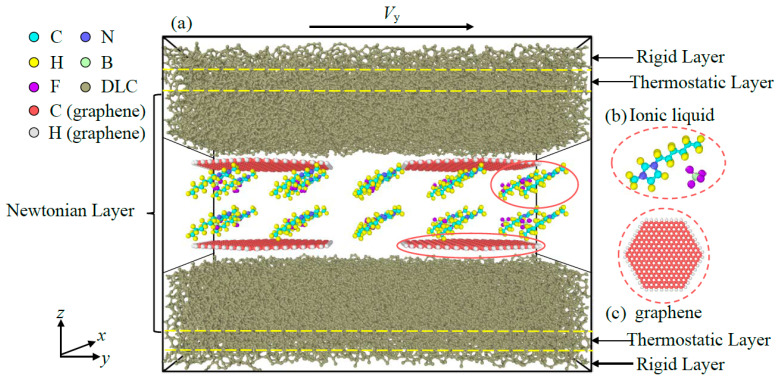
Atomic configuration of simulation model (**a**) schematic diagram of IL-based lubricants; (**b**) molecular structure model of 1-hexyl-3-methylimidazolium tetrafluoroborate and (**c**) graphene.

The atomic configuration consists of two layers of DLC films with IL lubricants and graphene additives located between the interfaces, as shown in Figure 1. The regular hexagonal graphene flakes with 19.8 Å in side length are added as additives into the lubricants. The size of each DLC film is about 44.4 × 99.8 × 22.4 Å^3^. The IL is consisted of the cation (1-hexyl-3-methylimidazolium, chemical formula: C_10_H_19_N_2_^+^) and the anion (tetrafluoroborate, chemical formula: BF_4_^−^) (as shown in Figure 1a). The atomic structure of pristine graphene consists of sp^2^ carbon (C) atoms arranged in the hexagonal symmetry and H atoms passivating its edge (Figure 1b).

The amorphous structure of diamond-like carbon film is obtained via a melting-quenching technological simulation process; more details of this process is provided in previous works [32]. The fraction of sp^3^C atoms in obtained DLC films is about 23%. The molecular structure of ionic liquids is optimized via the OPLS-AA force field.

Each DLC substrates were divided into three layers from the outermost to the interface near to the friction region, defined as rigid layer, thermostatic layer, and Newtonian layer, respectively, in the configuration. The outermost layer with a thickness about 4 Å was defined as rigid layer in which atoms’ relative position remain unchanged in sliding. The upper rigid layer was employed to apply the normal force and the sliding velocity. The lower rigid layer was frozen in a fixed position to realize the relative sliding. The thermostatic layer which is near to the rigid layer was employed to control the system temperature at about 300 K by adjusting the linear velocity of atoms. The remaining layer which is adjacent to the friction region is defined as Newtonian layer, in which mechanical and kinematic behavior of the atoms satisfy the second law of Newton.

The friction simulation process consists of three stages. In the first stage, the entire system is relaxed at 300 K to eliminate the residual stress of DLC and randomly distribute the lubricant molecules. In the subsequent stage, a load is applied to the rigid layer of the upper DLC film along *z*-direction by adding a uniform force to each of its atom. For the final stage, a sliding velocity along the *y*-direction was imposed on the rigid layer of the upper DLC film to realize relative sliding between two substrates. The boundary conditions are set as periodic along the *x*-direction and *y*-directions, while *z*-direction boundary condition is non-periodic. The time step in the simulation is set as 0.0002 ps; the whole simulation process is conducted for 400 ps to ensure credible friction results.

The molecular dynamics simulation is carried out by large-scale atomic/molecular massively parallel simulator (Lammps). The open source software OVITO 3.8.0 [33] was used to visualize the friction configurations. The interactions among C atoms of DLC and atoms in graphene were described by AIREBO potential, which is extensively employed to capture the formation and fracture behavior of chemical bonds between C and H atoms. The Lennard–Jones potential was employed to describe the interaction between the atoms in ILs and the atoms in the rest of simulation systems, wherein the partial parameters have been verified and widely used in the previous literature [34]. The other parameters of potential function were calculated using the Lorentz–Berthelot formula detailed in the previous literature [35].

In the simulation, considerations are given to different factors, such as normal stress *F*_n_ (2, 5 and 10 GPa); the corresponding normal forces, which are 44 nN, 220 nN, and 440 nN, respectively; IL amounts *N*_i_ (10, 20 and 40), graphene amounts *N*_g_ (2, 4 and 6); surface roughness *R* (1, 3 and 5 Å); and sliding velocity *V*_y_ (1, 1.5 and 2 Å/ps), to investigate the tribology behaviors. The substrate rough surface was obtained by deleting the partial atoms adjacent to friction region according to the following sine function.

(1) z<R×sin(0.25×y)+54&&z>R×sin(0.25×(y+6))+19.5

where, *z* is the coordinate of the atoms in the direction of normal stress, *R* the amplitude of roughness, and *y* controls the distance between two rough micro-convex bodies. The *F*_f_ is calculated by summing the tangential force along the sliding direction of the rigid layer in upper DLC. The molecular average translational kinetic energy formula, *E*_k_ = 3*k*_B_T/2 [36], was used to calculate the temperature (*T*) of the friction region. The coordination number of C atoms of DLC and graphene was calculated according to a cutoff length of about 1.9 Å, which corresponds to the value of the first minimum of the radial distribution function. The coordination number of C atoms with sp^2^ and sp^3^ hybrid states is three and four, respectively. The value of *f*_sp3_ was calculated by dividing the number of C atoms with the sp^3^ hybrid state by the total number of C atoms outside the rigid layer. The binding energy between multilayer graphene was calculated when two graphene sheets completely overlapped. The atomic stress tensor was calculated by multiplying the Voronoi stress tensor by an individual atom’s volume.

## 3. Results

### 3.1. Friction Behaviors with Different Normal Stress and Amounts of Graphene

**Figure 2:**
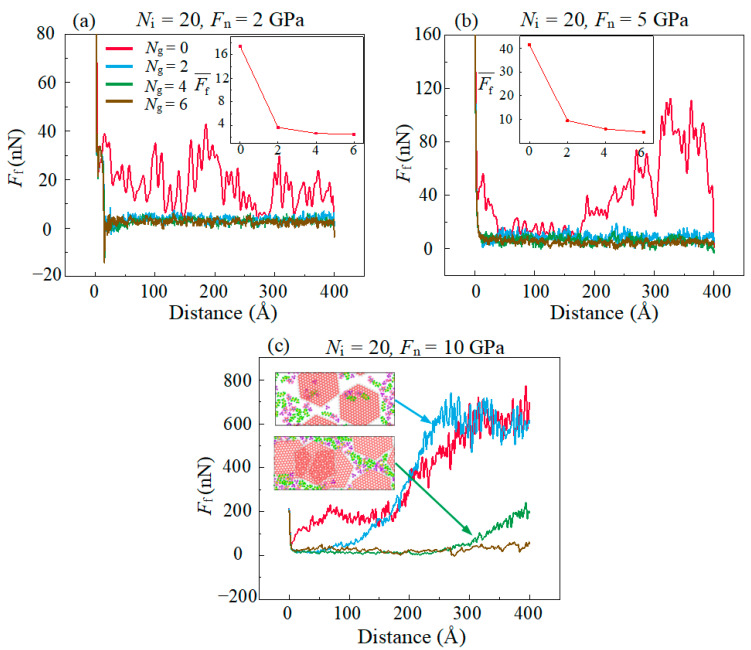
Evolution of the friction force *F*_f_ with the increase in the sliding distance in different normal stress *F*_n_ and graphene amounts *N*_g_. The illustration shown in the inset of (**a**,**b**) depicts the average value of *F*_f_ during the whole sliding process. The snapshots shown in the inset of (**c**) are the cross-sectional view of the lubricated area.

Figure 2 shows the effect of normal stress (*F*_n_) and amounts of graphene (*N*_g_) on friction force (*F*_f_). The short period (approximately 5 ps) after the beginning of sliding is defined as the running-in stage. In this stage, *F*_f_ is at a large value and then drops rapidly, which is due to the relative sliding of the friction pair required to overcome a large initial resistance. The running-in stage is followed by the stable friction stage. It can be seen from the illustration in Figure 1 that compared with pure ionic liquids (ILs), the Ff decreases significantly after adding graphene, with the maximum reduction of about 88%. This shows that graphene as an additive can enhance the friction reduction performance of IL lubricants.

It can be observed that *F*_f_ is kept at a small value in the whole sliding process in the condition of *F*_n_ ≤ 5 GPa. Such a phenomenon can be explained by the fact that the upper and lower layers of DLC are completely separated via composite lubricating film and there is no bonded interaction at the interface which resulted in extremely minute interfacial adhesion. When *F*_n_ continues to increase to 10 GPa, *F*_f_ increases more rapidly and the *F*_f_ at the final state is consistent with that of without graphene. 

**Figure 3:**
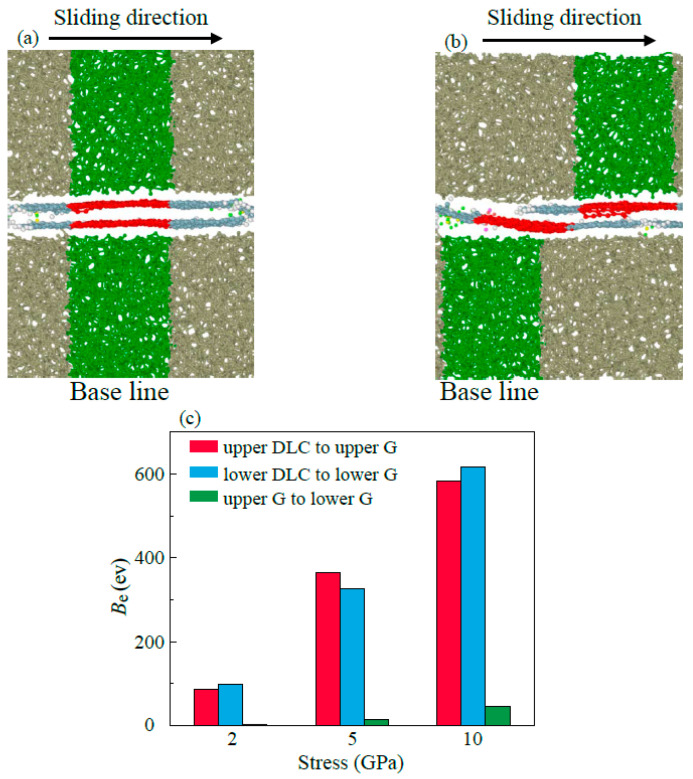
Snapshots of (**a**) initial (**b**) final sliding states lubricated by the multilayer graphene hiding the ionic liquid molecules. The green base line is used to visualize the relative motion between the DLC substrate and the red base line is used to visualize the relative motion between the multilayer graphene. (**c**) is the bonding energy *B*_e_ between two graphene layers and between an upper or lower graphene and the DLC substrate on the same side, respectively, when the two layers of graphene are completely overlapped.

When the monolayer graphene is present (*N*_g_ = 2, as shown in Figure 2c), the *F*_f_ increases rapidly at a large distance in the case of high *F*_n_. For the multilayer graphene (*N*_g_ = 4, as shown in Figure 2c), the *F*_f_ is rather low at the whole sliding process. At low *F*_n_, the *F*_f_ has a lower average value in the whole sliding process with the increase in *N*_g_, as shown in the inset of Figure 1a,b. The graphene with multilayer structure exhibit better lubrication performance, which is consistent with the previous literature [37]. The lower *F*_f_ with multilayer graphene can be explained by the super-low interlayer binding energy (*B*_e_) between graphene layers. The *B*_e_ between friction interfaces was calculated, as shown in Figure 3c. The graphene layer has a strong *B*_e_ with the upper and lower DLC substrates which makes the graphene move synchronously with the same-side substrate. The weak interlayer *B*_e_ between graphene leads to the relative movement between the overlapped graphene layers, as shown in Figure 3a,b. The relative position of the bottom graphene layer with the lower DLC substrate remains unchanged, but the top graphene layer moves along with the upper DLC substrate during the sliding process, which coincides with a previous study [38]. This is ascribed to the Be between DLC substrate and graphene layers which is larger than the one between the multilayer graphene. Therefore, the friction interface is transferred to the interface with lower *B*_e_, which leads to the reduction in friction lubricated by multilayer graphene during the friction simulations. Meanwhile, the low *B*_e_ between multilayer graphene ensures that the structure is more difficult to rupture [39], and the multilayer graphene can adapt to continuous operation under high *F*_n_ [40,41].

**Figure 4:**
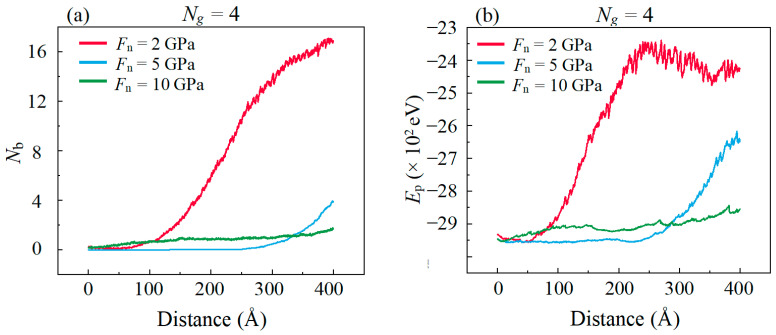
Evolution of (**a**) the covalent bonds *N*_b_ between graphene layer and DLC films (**b**) the average potential energy *P*_e_ of a graphene with the increase in the sliding distance in different *F*_n_.

In the simulation, it is observed that the layered structure of graphene is ruptured during the sliding process when *F*_n_ = 10 GPa. The C atoms in the graphene layer reach a higher potential energy state after the breaking of the C–C covalent bonds. Hence, we calculate the average potential energy (*P*_e_) of C atoms in the graphene layers shown in Figure 4a to validate the rupture state of graphene. It is observed that the *F*_f_ (Figure 2c) increases almost simultaneously with the commencement of the rupture of the graphene layer. This increase is attributed to the high interfacial adhesion caused by the formation of covalent bonds after the graphene rupture to produce dangling bonds. Figure 4b shows the amounts of interfacial C–C bonds (*N*_b_) between the graphene layer and the DLC film;, the simultaneously increase in *N*_b_ with *B*_e_ is because of the dangling bonds produced by the bond breaking in the graphene layer and which form the C–C covalent bonds with the C atoms in DLC film across the contact interface. Meanwhile, the large amounts of *N*_b_ causes a sufficient adhesion force and thus lead to a remarkable increase in sliding resistant force. This result demonstrates that the good lubrication performance of graphene in low *F*_n_ disappeared in high *F*_n_ and this is ascribed to the rupture of graphene.

**Figure 5:**
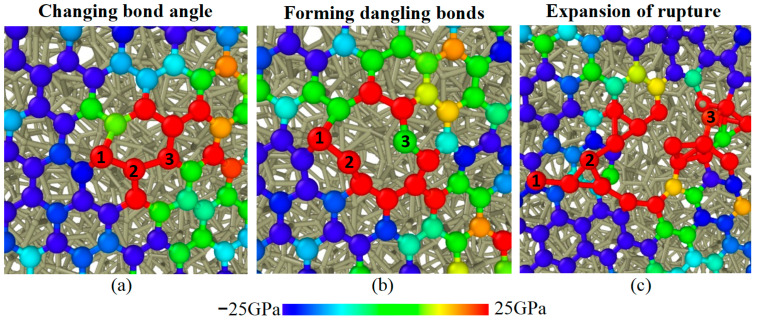
Evolution of local structure of graphene during (**a**) initial, (**b**) intermediate, and (**c**) final sliding states when *N*_g_ = 2 and *F*_n_ = 10 GPa. The sites subjected to tensile stress is visualized in red color, while the sites subjected to compressive stress is visualized in blue color. The numerical identifiers in atom are used to visualize structural changes.

Multilayer graphene shows a persistent lubrication performance compared to monolayer graphene, which demonstrates that the multilayer graphene structure has better mechanical properties during sliding. In order to figure out the rupture process of the graphene layer, the normal stress state of C atoms in the graphene layer on the sliding plane was calculated during sliding. The local structural evolution of the graphene layer is shown in Figure 5. It can be observed that the hexagonal structure remains intact but has changed bond angles in the sites with high tensile stress, while the bond angles remain intact in the sites with compressive stress in the initial sliding state. The bond of C atoms with preternatural bond angles is firstly breaking and producing dangling bonds in an intermediate state. Then, the local portion of bond fracture is expanding during sliding. The evolution of configuration determines that the rupture process of the graphene layer originates from the excessive tensile stress sites in the graphene layer.

**Figure 6:**
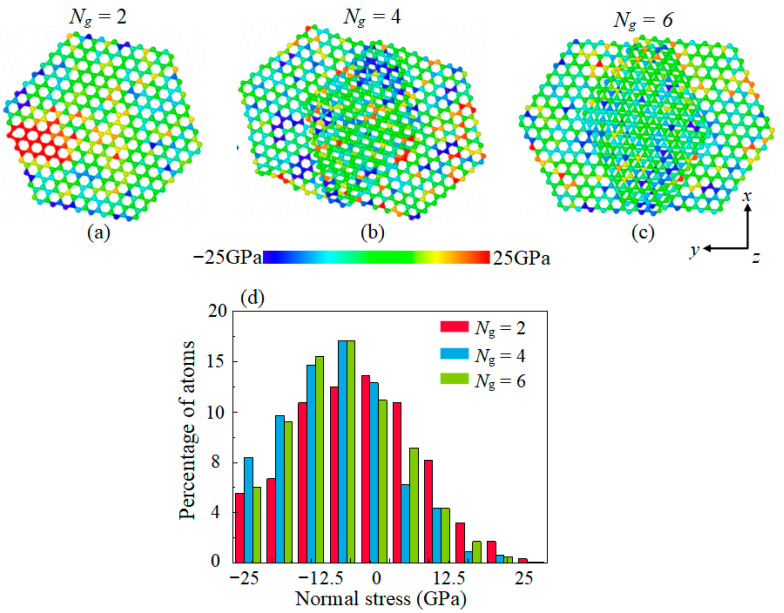
The normal stress distribution of C atoms in intermediate sliding state with different amounts of graphene when *F*_n_ = 10 GPa. (**a**–**c**) The sites subjected to tensile stress are visualized in red color, while the sites subjected to compressive stress are visualized in blue color; (**d**) Histogram of normal stress of C atom of graphene on the *x–y* plane, the normal stress is divided into 10 intervals in the range of −25 GPa to 25 GPa, and the proportion of atoms in each interval to all atoms in graphene is counted.

To further quantitatively investigate the effect of layer structure on rupture behavior of graphene, we performed normal stress sites analysis for different layered graphene structures. Figure 6a–c shows the *x*–*y* plane normal stress distribution of C atoms when *F*_n_ = 10 GPa. For *N*_g_ = 2, we snapshot a piece of graphene with a monolayer structure as a reference, and snapshot a part of the graphene overlapped with multilayer structure in the case of *N*_g_ *≥* 4. It can be observed that there are more C atoms subjected to high tensile stress in the monolayer graphene. The distribution of normal stress of C atoms is almost identical for the multilayer structure between *N*_g_ = 4 and *N*_g_ = 6; most of the C atoms in the graphene layer are subjected to compressive stress and only few atoms are under tensile stress. To quantify this, we plotted the histogram of normal stress of C atoms with different amounts of graphene. The histogram has larger quantity at low-magnitude normal stress and lower quantity at high-magnitude normal stress. It should be noted that the highest frequency of the high-magnitude tensile stress range is in the case of *N*_g_ = 2, while the lowest frequency is in the case of *N*_g_ = 6. In both cases of *N*_g_ = 4 and *N*_g_ = 6, the distribution frequency of atomic normal stress is almost identical. This is because all graphene exist in lubricant as overlapping-layer structure in the two cases. The above phenomenon is consistent with the normal stress distribution of C atoms with different layered structure of the graphene, as shown in Figure 6a–c. It confirms the weakening effect of percentages of tensile stress sites by multilayer structure. Above results demonstrates that the multilayer graphene has better mechanical property compared to the monolayer graphene. Hence, the multilayer graphene has better anti-rupture performance. 

### 3.2. Friction Behaviors with Different Amounts of Ionic Liquids 

**Figure 7:**
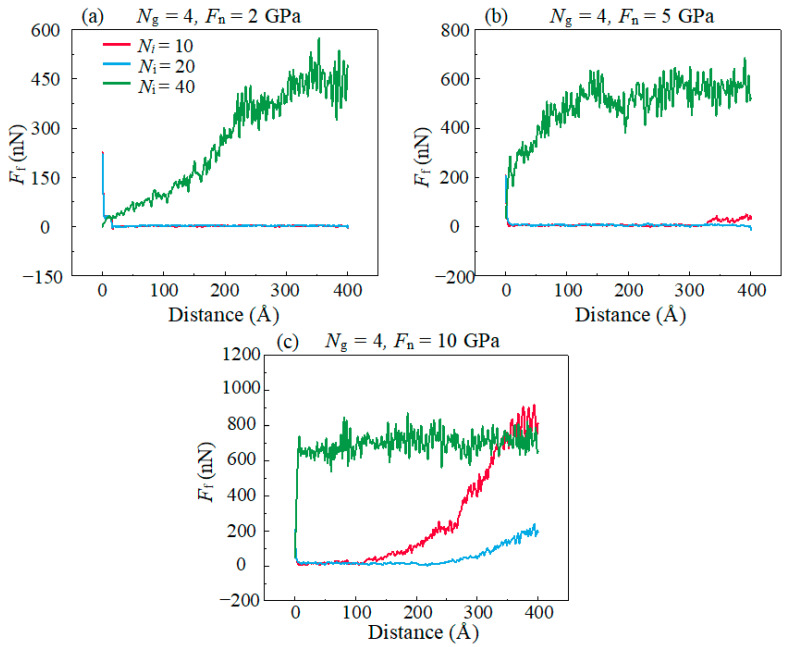
(**a**–**c**) Evolution of the *F*_f_ with the increase in the sliding distance in different *F*_n_ and amounts of ILs.

Figure 7 shows the effects of *F*_n_ and ionic liquids amounts (*N*_i_) on the performance of IL-based lubricants by employing constant graphene amounts (*N*_g_ = 4). In the case of small amounts of IL (*N*_i_ ≤ 20), the *F*_f_ remains at a small value after sliding which is attributed to the separation effect of IL-based lubricants on the DLC friction pairs and low interfacial adhesion between the friction interface. 

For the three lubrication conditions of *N*_i_ = 10, 20 and 40, we define them as “insufficient”, “appropriate” and “excessive” amounts, respectively. Different *N*_i_ represent different thicknesses of the lubricating film. When the *N*_i_ ≤ 20 and *F*_n_ = 10 GPa, the IL-based lubricants show more persistent lubrication performance with the amounts of IL increasing from insufficient to appropriate; this is because the increase in *N*_i_ enhance the dispersion of graphene. On the other hand, in the case of excessive amounts of ILs (*N*_i_ = 40), the *F*_f_ increases rapidly after sliding a short distance and the increasing rate of *F*_f_ is more obvious under higher *F*_n_. The high *F*_f_ demonstrates that excessive amounts of ILs may result in the immediate invalidation of the performance of IL-based lubricants.

In previous studies, the decrease in fluidity of graphene caused by sheets aggregation was used to interpret the poor lubrication at high graphene concentration [42]. In order to verify this index, the mean square displacement (MSD) curves of graphene are calculated at different amounts of ILs. However, the results show that the amounts of ILs have almost no effect on the MSD of graphene under the given conditions. The decrease in fluidity of graphene is inappropriate to interpret the high *F*_f_ at high graphene concentration in our research.

**Figure 8:**
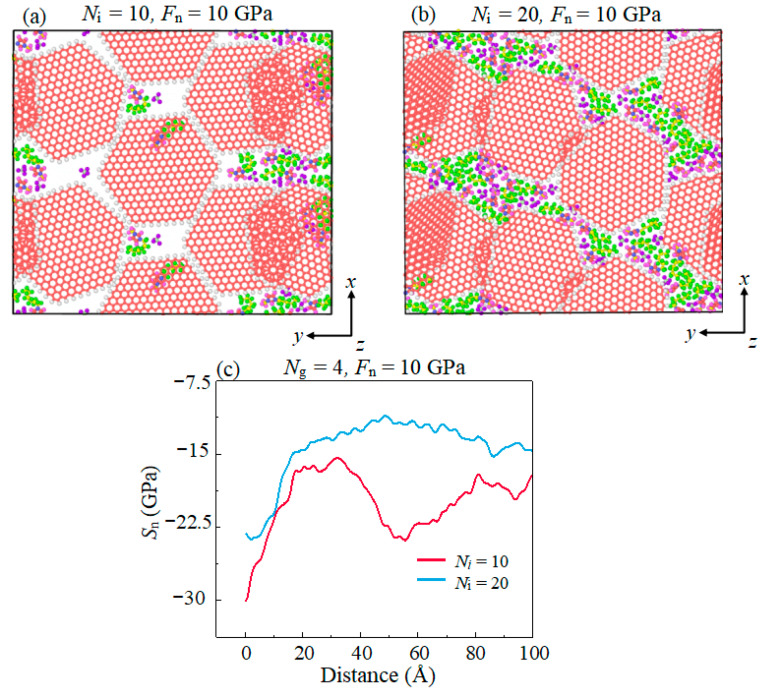
The cross-sectional view of the lubrication area with (**a**) *N*_i_ = 10, (**b**) *N*_i_ = 20, and (**c**) the average normal stress *S*_n_ of the C atom in graphene layer on the *z*-direction with different *N_i_*.

In this work, the normal stress state of C atoms in the graphene was calculated to explain this high *F*_f_, as shown in Figure 8a,b. A large amount of space voids are formed in the IL-based lubricating film due to insufficient lubricants in the case of *N*_i_ = 10. This loose configuration of lubrication film comprises most of the load acting on the lubricating film loaded by the graphene layer which make the stress on the graphene greater than the theoretical value (*F*_n_ = 10 GPa). However, for *N*_i_ = 20, this IL-based lubricating film is dense during sliding simulation which makes the stress on graphene closer to the theoretical value. The average normal stress (*S*_n_) value of the C atom in the graphene layer on the *z*-direction was shown in Figure 8c. Under the same *F*_n_, the *S*_n_ of graphene decreases with the increase in *N*_i_ in the sliding process. The increased amounts of IL is responsible for the normal stress on the graphene, which guarantees the stability of the graphene structure.

**Figure 9:**
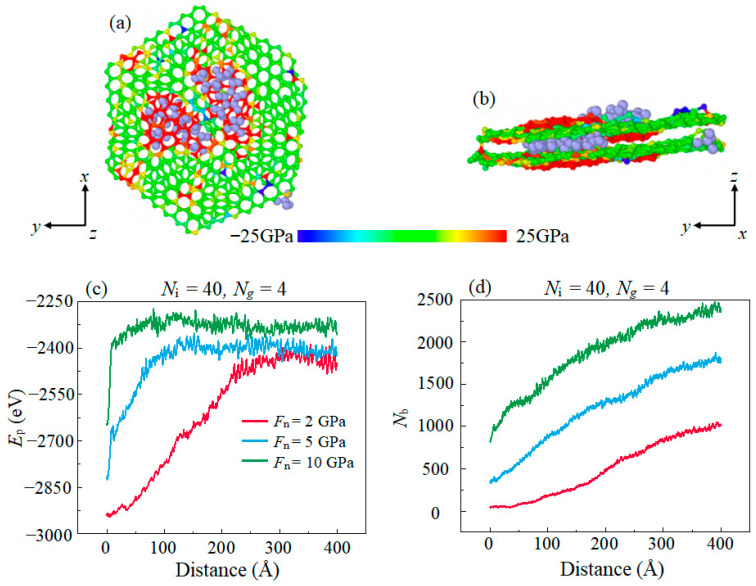
The snapshot of graphene layer and ILs in sliding process on (**a**) top view and (**b**) side view. The sites subjected to tensile stress are visualized in red color, while the sites subjected to compressive stress are visualized in blue color. (**c**) shows the evolution of potential energy *E*_p_ of a graphene with different *F*_n_, and (**d**) demonstrates the evolution of the amounts of interfacial C–C bonds between graphene and DLC films *N*_b_ with different *F*_n_.

In the case of excess amounts of IL (*N*_i_ = 40), the *F*_f_ increases sharply in the initial stage of sliding. Figure 9c shows that the total potential energy (*P*_e_) of graphene starts to increase remarkably in the initial stage of sliding. The increase in the *P*_e_ of graphene is due to the transition of C atoms from the sp^2^ hybridized state to sp^3^ hybridized state which indicates that the regular hexagonal structure of graphene has been ruptured during sliding. And the process of graphene structural rupture is shortened with the increase in stress. Figure 9d shows that the amounts of interfacial C–C bonds between the graphene layer and DLC films increase during sliding. This is because the free carbon atoms in the ruptured graphene layer form covalent bonds with the C atoms of the DLC films, which leads to the increase in the adhesion interaction between the contact interfaces, thus increasing the *F*_f_. To investigate the mechanism of the invalidation of lubricating performance caused by excess ILs, the stress state of graphene during sliding was calculated, as shown in Figure 9a,b. It can be observed that hard IL molecules are sandwiched between the dual surfaces of the graphene layer in simulation in the case of excess ILs, which is not the case in small amounts of IL. It forms a structure similar to a three-body abrasive wear structure and the surface of graphene is no longer flat, which weakens its mechanical strength. Parts of the graphene in contact with the IL molecules undertake high normal stress due to the local concentration of the contact stress. The stress of IL molecules on the surface of the graphene is larger than the crushing stress of the graphene layer, which leads to rapid damage of the graphene structure. Graphene forms a three-body abrasive wear structure with ILs in an over-thick IL lubrication film, which leads to the decline of its lubrication performance. This result is consistent with previous research [43], and also explains the reason for the decline in lubrication performance.

### 3.3. Friction Behaviors with Different Surface Roughness

**Figure 10:**
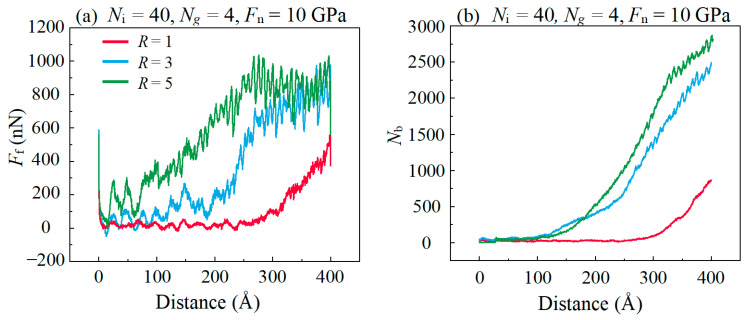
Evolution of (**a**) the friction force *F*_f_ and (**b**) interfacial C–C bonds between graphene layer and DLC films *N*_b_ with the increase in the sliding distance in different *R*.

**Figure 11:**
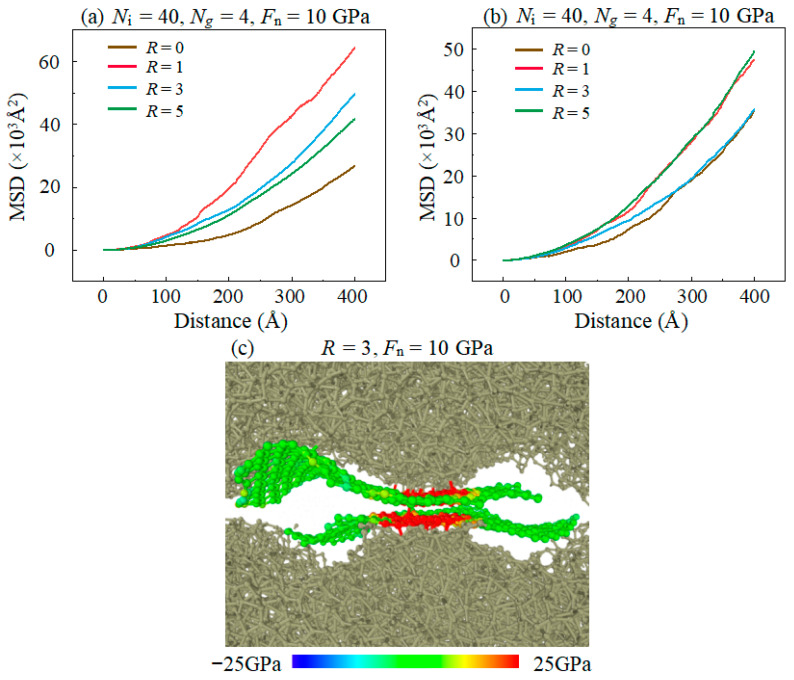
Evolution of the mean square displacement of (**a**) graphene, (**b**) IL with the increase in the sliding distance in different surface roughness. (**c**) is the local atomic configuration of the tribological interface of graphene layers in which ionic liquid molecules are hidden. The sites subjected to tensile stress are visualized in red color, while the sites subjected to compressive stress are visualized in blue color.

The effect of substrate surface roughness on the lubrication performance of IL-based lubricants were studied under the condition of constant amounts of ILs and graphene layers, as shown in Figure 10a. In the presence of surface roughness, the *F*_f_ keeps a relatively small value at the distance ≤ 100 Å. This is because graphene covers the rough surface to prevent the direct contact of the roughness bearing points and there is almost no chemical bond formation between graphene layers, as shown in Figure 11c. It helps the composite lubricant to still maintain its lubrication effect for a short time under large roughness. The *F*_f_ rapidly increases at a large distance, and the *F*_f_ in the condition of *R* ≥ 3 is much higher than in the condition of R = 1 at the distance ≥ 300 Å. This shows that the lubrication performance of a compound lubricant is sensitive to surface roughness when other experimental conditions are kept consistent, which is caused by a variety of effect factors. On one hand, the micro-convex structures of larger surface roughness induces a contact of higher local stress of graphene with this region, as shown in Figure 11c. And excessive stress concentration can easily lead to the rupture of the graphene structure. On the other hand, the animation of the simulation demonstrates that graphene is more prone to wrinkle and fold during the sliding process under rough substrate conditions, which leads to the possibility of structural damage of the graphene layer. In the case of a large surface roughness (*R* ≥ 3), the upper and lower surfaces of the DLC can contact directly and form a large number of covalent bonds, as shown in Figure 10b. These covalent bonds can greatly enhance the interfacial adhesion and result in high F_f_. Hence, in the case of different roughness, the number of C–C covalent bonds between lubrication interfaces is the leading factor affecting the different F_f_.

The surface roughness will also affect the fluidity of lubricants. The MSD of graphene increases obviously when the surface changes from smooth to roughness, as shown in Figure 11a. This is because the graphene layer in contact with the micro-convex body undertakes large *F*_n_ and forms a covalent bond with the substrate which makes graphene have a great adhesion to the substrate, while there is almost no covalent bonds between graphene layers due to the strong van der Waals force, as shown in Figure 11c. Hence, the graphene layer is almost adsorbed on the rough substrate and moves synchronously with the DLC in the upper layer. It prevents direct contact between the micro-convex structures and keeps the *F*_f_ at a relatively small value during sliding. Figure 11b shows that the MSD of ILs also increases when the surface changes from smooth to rough. It is indicated that roughness enhances the fluidity of ILs which has a positive effect on reducing friction. However, the *F*_f_ is still higher in the case of surface roughness than in the case of smooth plane; it indicates that the separation of the graphene layers to DLC pairs and the enhancement of the diffusion performance of compound lubricant weaken the *F*_f_ to some extent, but the bonding between DLC substrates still plays a dominant role.

### 3.4. Friction Behaviors with Different Sliding Velocity

**Figure 12:**
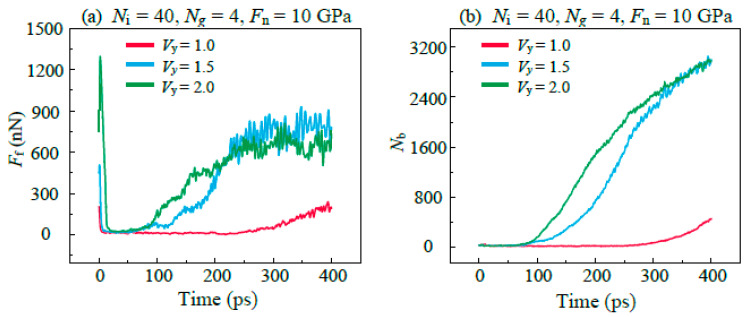
Evolution of (**a**) the friction force *F*_f_ and (**b**) interfacial C–C bonds between graphene layer and DLC films *N*_b_ with the increase in the sliding distance in different *V*_y_.

The effect of sliding velocity (*V*_y_) on the lubrication performance of IL-based lubricants was studied under the condition of constant *N*_i_ and graphene layers, as shown in Figure 12a. The *F*_f_ has a short process of rising and falling at the beginning of sliding which corresponds to the activation of lubricants flow under shear stress, and the amplitude increases with the *V*_y_. At a low sliding speed (*V*_y_ = 1 Å/ps), IL-based lubricants can maintain more persistent lubrication performance. The analysis of the bonding between graphene and DLC shows that the amounts of covalent bonds between the interfaces increases significantly with the sliding velocity. This is because high sliding velocity induces the formation of wrinkles in the graphene, which can significantly drop the load-carrying capacity (critical value of graphene failure) by almost an order of magnitude [27]. Therefore, the graphene is more prone to rupture and resulting failure of lubrication at high *V*_y_.

**Figure 13:**
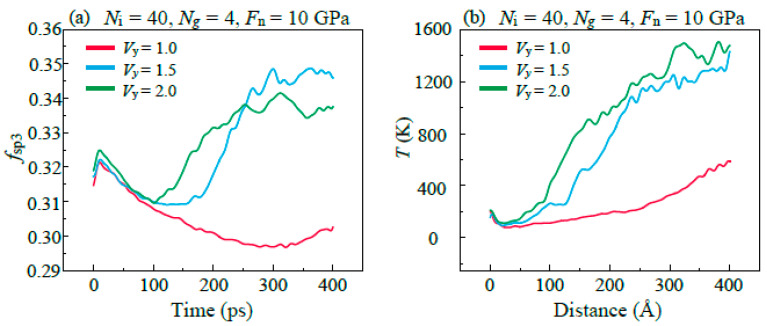
Evolution of (**a**) the fraction of C atoms with sp^3^ hybrid state *f*_sp3_ with the increase in sliding time, and (**b**) temperature of friction area with the increase in the sliding distance in different *V*_y_.

After the stabilization of *F*_f_ in high sliding velocity (*V*_y_ ≥ 1.5 Å/ps), the friction results show that the increase in velocity reduces the *F*_f_ in the stable stage, which can be explained by the high friction temperature caused by high speed. The failure of lubricant leads to the direct formation of C–C covalent bonds between the upper and lower DLC films and results in mechanical mixing at the contact interface. In this condition, the *F*_f_ depends on the composition and structure of the mixing interface. Figure 13a shows the evolution of the fraction of sp^3^C bonding atoms. In the early stage of friction, the decrease in *f*_sp3_ corresponds to the sp^3^–sp^2^ transitions (graphitization) in the DLC films which is induced by the high temperature in the friction region, as shown in Figure 13b. The result shows that a high sliding velocity leads to a higher degree of graphitization. In the next stage, the increase in *f*_sp3_ is because the sp^2^-bonded C atoms in graphene bonded with C atoms in DLC films to form the sp^3^-bonded C atoms during the process of structural damage of the graphene. After the stabilization of *f*_sp3_, the *f*_ps3_ in the case of *V*_y_ = 2 Å/ps is lower than that of *V*_y_ = 1.5 Å/ps. This is because the high temperature in the friction region caused by high *V*_y_ promotes the graphitization of C atoms at the friction interface. It has been demonstrated by many previous studies that high sp^2^C content can lead to smaller *F*_f_. The graphitization caused by high temperature results in the easy-shear properties and loss of the structural mechanical strength of DLC friction interfaces [44], and thus contributes to the reduction in *F*_f_ in the stable stage.

**Figure 14:**
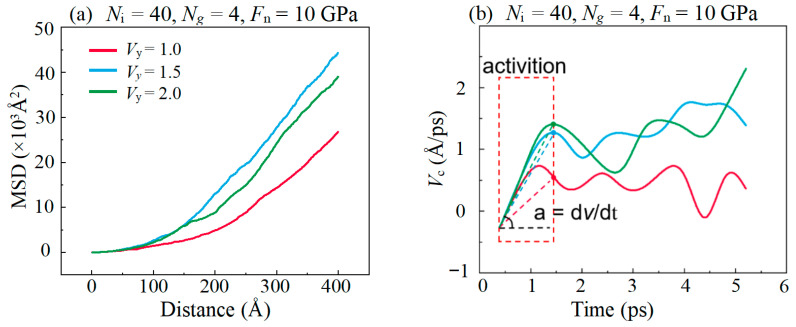
Evolution of the (**a**) mean square displacement with the increase in distance and (**b**) the centroid velocity *V*_c_ of lubricant molecules with the increase in sliding time under different sliding velocity.

The increase in speed enhances the fluidity of the IL-based lubricants, as shown in Figure 14a. This is because graphene is more likely to form wrinkles and curl the surface at high *V*_y_, which makes it easier for the graphene to promote the common sliding of IL molecules along the sliding direction, thus enhancing the fluidity of the lubricants. In the initial stage of friction (Time < 1 ps), the flow of lubricant is activated due to the shear stress of the upper DLC film, as shown in Figure 14b. The velocity of the center of mass of the lubricant molecules reaches a stable value within 1 ps, and the increase in the sliding velocity increases this stable value. It indicates that the lubricant molecules at high sliding velocity need a greater acceleration at the startup stage, so this requires the upper DLC film to provide a greater initial *F*_f_ to activate the lubricant films at the initial stage of friction. As the velocity of the lubricant molecules reaches a stable level, the *F_f_* decreases to a stable value.

## 4. Discussion

Graphene is an excellent IL lubricant additive owing to its high load-carrying capacity and its ability to separate the surface of the friction pairs. At low *F*_n_, the lubricating performance of the graphene-added lubricants is superior to that of pure IL lubricants, which is due to the fact that intact graphene prevents direct contact of the friction pairs interface. As the *F*_n_ increases, graphene is subjected to high stress to make its structure unstable and C atoms with high chemical activity appear in the graphene. These atoms form covalent bonds with the C atoms in the DLC to rupture the 2D structure of graphene and cause an increase in *F*_f_. The simulation experiments show that the lubricant is ineffective due to the structural destruction of the graphene when *F*_f_ is large enough, as evidenced by the comparison of tribological properties with pure ILs at high *F*_n_.

Previous experiments have shown that the worse tribological performance of pure ILs lubricants is due to the poor load-carrying capacity in high *F*_n_ [15,45]. For the composite lubrication film with graphene as the additive, they can greatly improve the load-carrying capacity and obtain longer anti-wear life than IL lubricants. Meanwhile, the addition of graphene additives will affect the continuity of the lubricant. Relative excess graphene has a negative impact on the continuity of ILs lubricant, as shown in Figure 8a. Graphene separates the IL lubricants in several nonadjacent spaces, which form several void spaces in the lubricating film. The negative effects of film continuity will lead to the reduction in lubricating performance, which will eventually lead to the increase in friction. The effect of film continuity is positive for rough DLC substrates. The IL film will not be able to withstand plastic deformation when the stress is directly and vertically applied to the sliding direction. However, graphene can withstand the direct contact between the contact surfaces, which ensures that the lubricating film always maintain a continuous and stable lubrication performance.

Previous studies have shown that the performance of graphene as additives for IL lubricates is connected with the concentration and the presence of the layered structure of graphene. He et al. found that low concentrations of graphene oxide sheets as additives for lubricants has poor lubricating effects [46]. Saurin et al. studied the effect of graphene with different number of layers as an additive on the performance of IL lubricants. Graphene with a low number of layers (1–2 layers) easily forms grindable polymers with ILs, resulting in the degradation of lubricating properties. Graphene with more layers (2–10 layers) has excellent properties as an additive under various friction conditions [28]. We demonstrate that this difference is due to the interlayer easy-shear ability of multilayer graphene, whose realization requires that the binding energy between graphene plane hexagons is lower than that between the substrate and graphene. When shear force is applied to the graphene, multilayer graphene will move relatively between layers, thus reducing the coefficient of friction. Moreover, the superposition behavior of graphene makes its structure more stable and gives it longer anti-wear life than monolayer graphene. The relief of tensile stress by multilayer graphene observed in this research must be further studied in the future.

Under all the conditions given in this study, the addition of graphene resulted in a significant reduction in *F*_f_, which was ensured by the synergistic effect of graphene and ionic liquids. On one hand, previous research have shown that graphene is easily torn by the frictional substrate [47], and the lubricating behavior of the ionic liquid releases the lateral stress of the frictional substrate on graphene, thus ensuring its structural integrity. On the other hand, some previous studies have also shown that the agglomeration behavior of ionic liquids under high pressure leads to a shift from fluid to boundary lubrication [48]. Graphene as additives attenuate the agglomeration behavior of ILs, thus enhancing the load-bearing capacity and ensuring high lubrication performance.

## 5. Conclusions

In this paper, an atomic configuration comprising two DLC substrates and IL lubricants with graphene additives was constructed to evaluate the tribological behavior of the lubricants using MD simulation. Through the systematical analysis of the evolution of friction interface, dynamic trajectory of the lubricant, and stress state of the additives and friction property, we arrived at the the following conclusions:

(1) Graphene as lubricant additives can improve the lubrication performance of ILs at all given conditions. The reduction reach up to 88%. The improved tribological properties of composite lubricants are attributed to the fact that intact graphene prevents direct contact of the friction pairs interface, but fail under high normal stress due to structure rupture.
(2) Multilayer graphene has better friction-reducing performance and better friction durability compared to monolayer structure. The antifriction performance is ascribed to the easy-shear property between the multilayer graphene layers with super-low binding energy, which is 5% of the binding energy between the DLC and the graphene. The friction durability is due to the decrease in the percentage of high tensile stress sites which ensures that the multilayer graphene has a more stable structure in lubrication.
(3) The lubricating performance of the compound lubricating oil is sensitive to *N*_i_. Void space occurring under insufficient *N*_i_ in lubrication film makes the *F*_n_ acting on graphene higher than the theoretical value, which then makes graphene prone to rupturing under the same theoretical pressure. Over-thick IL film and graphene form a three-body abrasive wear structure with stress concentration in the graphene, and eventually lead to degradation of tribological performance of the compound lubricant.
(4) Both the rough surface of the substrate and the high sliding speed affects the lubricating properties of the lubricant, which is caused by the excessively concentrated stress in the graphene plane, promoting the bond formations between graphene and DLC substrates. In addition, it is found that graphitization caused by high temperature effectively reduces friction in the stable stage in high speed.
(5) The results of this study explain the basic friction mechanism of graphene additives in ionic liquid lubricants, and provide a reference for the development of new fluid lubricants by adjusting the content of graphene, normal stress and the thickness of ionic liquid lubrication film to meet potential applications.

## Data Availability

Data will be made available on request.
